# Improvement of Biocatalysts for Industrial and Environmental Purposes by Saturation Mutagenesis

**DOI:** 10.3390/biom3040778

**Published:** 2013-10-08

**Authors:** Francesca Valetti, Gianfranco Gilardi

**Affiliations:** Department of Life Sciences and Systems Biology, University of Torino, via Accademia Albertina 13, Torino 10123, Italy

**Keywords:** biocatalysis, directed evolution, synthetic biology, protein engineering, industrial biotechnology, bioremediation, fine chemistry, saturation mutagenesis, screening methods

## Abstract

Laboratory evolution techniques are becoming increasingly widespread among protein engineers for the development of novel and designed biocatalysts. The palette of different approaches ranges from complete randomized strategies to rational and structure-guided mutagenesis, with a wide variety of costs, impacts, drawbacks and relevance to biotechnology. A technique that convincingly compromises the extremes of fully randomized *vs.* rational mutagenesis, with a high benefit/cost ratio, is saturation mutagenesis. Here we will present and discuss this approach in its many facets, also tackling the issue of randomization, statistical evaluation of library completeness and throughput efficiency of screening methods. Successful recent applications covering different classes of enzymes will be presented referring to the literature and to research lines pursued in our group. The focus is put on saturation mutagenesis as a tool for designing novel biocatalysts specifically relevant to production of fine chemicals for improving bulk enzymes for industry and engineering technical enzymes involved in treatment of waste, detoxification and production of clean energy from renewable sources.

## 1. Introduction

Protein engineering allows exploration of mutational space under artificial evolutionary pressure and selection that could not be sampled by the natural environment of proteins. Advances in this field demonstrate how natural catalysts can be finely tuned to perform reactions that are new in terms of specificity [1,2,3], efficiency [4,5,6], stability of the enzyme, conditions [7,8] and chemistry of the reaction catalyzed [9,10,11]. Many of the biotechnological benefits of this “laboratory-driven evolution” have already been translated into practical applications, and many others can be foreseen to have a high impact in sustainable and innovative processes [12], environmental bioremediation, detoxification, and clean energy production [13,14,15].

New tools and strategies aiming at simplifying the experimental work required for a successful result in obtaining engineered enzymes are continuously being developed. The methods follow two main directions: the rational site-specific mutagenesis and the evolution-like random approach. Both are powerful but each suffer from different limitations in the performance of the outcome and in time necessary to achieve the results. The rational site-specific mutagenesis focuses on the mutation of one or more specific amino acids that are replaced with another residue. It needs to be supported by structural and functional data of the enzyme and it is frequently biased by the assumptions made by the researchers on the basis of previous knowledge. In this respect it might be less innovative and aim at less ambitious goals, although it remains a very precious strategy for testing hypothesis on the fine details and structural determinants of reaction mechanisms. Compared to the random evolution-like approach, it is less time consuming in the production of the mutants, but in the perspective of producing significantly improved biocatalysts for industrial applications, it often results in limited improvement of the desired property. Results are achieved through series of trial-and-error experiments that surely provide interesting data for theoretical speculation but that may require large amounts of time and resources. 

On the other hand, laboratory evolution is based on the selection of random mutants with the desired features. It is not limited by the availability of the 3D structure of the enzyme and it mimics in the lab the evolution process that in nature has led to the selection of the best natural catalysts available: the enzymes. This approach establishes methods to introduce random genetic diversity in libraries of mutants (variants) that include various implementations of mutagenic PCR, oligonucleotide-assisted mutagenesis and *in vitro* recombination under mutagenic conditions, including DNA shuffling [16] and several specific techniques such as ITCHY [17], RACHITT [18] SHIPREC [19] and many others that have been extensively reviewed [20,21,22,23,24,25]. The time consuming process of obtaining the randomly mutated library and the requirement for a high-throughput screening procedure for selection of the desired properties among thousands of clones, is the severe drawback of a very powerful technique that otherwise has the advantage of providing entirely novel landscapes of mutants [26,27].

A specific type of laboratory-evolution method is the “targeted random mutagenesis” method, also called “saturation mutagenesis” that focuses on specific “hot spots” for mutational variability or on critical residues identified by structural comparison and modeling methods. It applies site-saturation mutagenesis (SSM), *i.e.*, the systematic replacement of one amino acid at a chosen site with all alternative encoded amino acids, to explore the performance of each possible variant in terms of structural or functional features of the resulting mutated enzyme. SSM may be applied at random positions but more often it is based on the assumption that most mutations are deleterious or neutral, and therefore the construction of mutant libraries by random methods is inefficient. Since the enzyme properties that are pursued are mainly codified in a small part of the enzyme corresponding to the active site or structural portions known to modulate protein stability, a rational choice of the sites to be targeted is usually preferred. This approach allows fine-tuning of the catalytic properties, particularly when performed as a refinement step after directed evolution. In fact, in fully random techniques a trade-off between the selected property and the overall enzyme performance might put an apparent threshold to the optimization of the target property [28]. Therefore, saturation mutagenesis is a precious tool for exploring and widening the landscape of the enzyme properties and applications. The advantages lie in a compromise solution combining the positive features of the rational mutagenesis and the random approach followed by laboratory selection, with minimum or negligible additive effect on the drawbacks. This is becoming clear in the last few years due to the increasing number of successful results obtained. A particular relevance is given in literature to positive results of this approach applied to enzymes used in fine chemical synthesis, industrial processing and bioremediation. 

In order to improve the outputs and to obtain libraries with high abundance methods such as iterative Combinatorial Active Site Test (CAST) [29] and Iterative Saturation Mutagenesis (ISM) [30], all based on the same principle of SSM, were more recently implemented. 

The methodology for Site Saturation Mutagenesis, Iterative Saturation Mutagenesis and other innovative methods will be presented highlighting advantages *vs.* site specific and random mutagenesis. Technical details and implications will be discussed, also tackling the issues of randomization and statistical evaluation of the library completeness and throughput efficiency of screening methods. Examples of successful applications covering different enzyme classes will be presented, focusing on cases that are relevant for the production of fine chemicals as well as bulk enzymes for industry, treatment of wastes, detoxification of pollutants and xenobiotics, and production of clean energy from renewable sources.

## 2. Experimental

Different methodologies pertaining saturation mutagenesis, leading to libraries of mutants relevant in terms of their size with minimal screening efforts, will be illustrated in the following paragraphs. The choice of alternative approaches bears crucial implications and must be carefully considered. Following the pattern of single site saturation mutagenesis and extending the strategies to various multiple combinations, a range of protocols have been proposed and tested. These are described and discussed here, together with the statistical analysis of library coverage and screening methods specifically for the saturation mutagenesis approaches.

### 2.1. Strategies for the Generation of Libraries of Mutants

#### 2.1.1. Site Saturation Mutagenesis (SSM)

**Figure 1:**
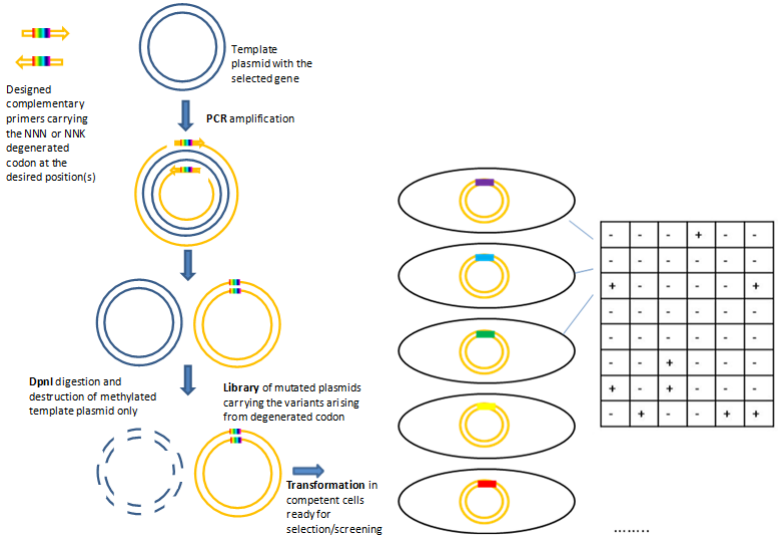
Scheme of site saturation mutagenesis approach following the QuikChange^TM^ kit.

The SSM libraries are usually generated with protocols that follow the commercially available QuikChange™ kit commercialized by Stratagene [31] or using equivalent in house procedures [30]. Mutagenic and complementary primers that carry the desired mutation (Figure 1) are used in a PCR reaction to amplify the plasmid with high fidelity and thus inserting the desired mutations. The position chosen for mutagenesis can be randomized with the codon NNN (where N is any nucleotide), or with a codon NNK (where K is either a T or a G) that can produce codons for all the 20 amino acids and a stop codon. Compared to NNN degeneration, NNK has the advantage that it will produce 32 variants instead of 64, reducing screening effort and inserting one stop codon instead of three. The mutagenic primers are designed with the targeted position in the middle and at least 15 non-mutated bases before and after the point of mutation. The PCR product is digested with DpnI, a restriction enzyme that recognizes and cleaves the methylated template DNA, while the non-methylated newly synthesized and mutated DNA strands are not recognized nor digested. The mutated nicked plasmid is transformed in highly competent *E. coli* strain DH5α or XL1-Blue.

#### 2.1.2. Iterative Saturation Mutagenesis (ISM)

**Figure 2:**
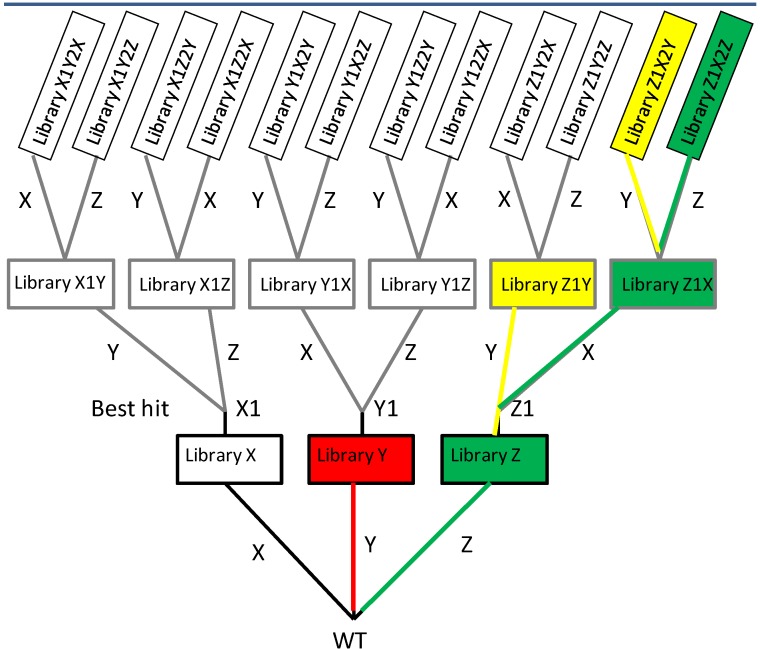
Scheme of iterative saturation mutagenesis showing the branching process and highlighting the productive pathway (in green), non-productive mutants that stop the process simplifying the screening procedure (in red). Highlighted in yellow are mutants produced with moderate to low improvement that can be discarded or reconsidered for further processing in a second phase.

The Iterative Saturation Mutagenesis (ISM) was proposed by Reetz and coworkers [30] and it combines, in an iterative manner, the SSM described above. While other strategies simply add mutations at rationally-chosen single sites by producing double or triple mutants that simply contain the positive mutation 1, 2, 3 *etc*., in the ISM approach, a few sites in the protein sequence are identified as crucial by means of structural data or modeling, requiring a partially rational approach as in SSM, but saturation mutagenesis is then applied at the chosen sites in a combinatorial pattern. The site can be represented by a single amino acid or by a few neighboring amino acids, ideally not more than three, keeping in mind that an increase in the number of variants will then require screening of a large number of clones. These sites are then mutated according to the saturation mutagenesis approach. The novelty of this approach resides in the iterative feature given by selecting the best hit of the library obtained at each target site. For example, assume sites X, Y, Z have been selected for mutagenesis. These sites will lead to three libraries X, Y, Z, each giving as best variant X1, Y1, and Z1. Saturation mutagenesis is applied at the respective other sites: X1 will be subjected to SSM at site Y, providing library X1Y, and at site Z, providing library X1Z, as shown in the scheme of Figure 2.

This branching process, iterated by applying SSM to a single site one or more times, can theoretically extend very quickly. For example, iterating each SSM at three sites results in 12 libraries as shown in Figure 2. In practice, non-productive branches will stop the process, as the example highlighted in red in Figure 2, while a pathway leading to synergistically improved mutants (*i.e*., not resulting from the simple sum of single mutations) can be efficiently defined, reducing the library size. The productive pathway is highlighted in green, while yellow is shown as another branch producing variants with limited improvement. Each new cycle of ISM maximizes the probability of obtaining additive and/or cooperative effects of newly introduced mutations, which optimize the fitness landscape in a defined region of protein sequence space. This is not the case when the best-hit mutant of each library is simply added to a double or multiple mutant, where the effect can be non additive or even detrimental to the desired protein property. ISM has been demonstrated to achieve impressive results especially in enhancing enantio-selectivity [32] and thermostability of enzymes. Notably, the ISM strategy was also tested on libraries that initially did not contain improved variants, by applying the iterative cycle even to inferior mutants as templates. This was done within the systematical testing of features of 24 alternative pathways to improved variants of a biocatalyst (epoxide hydrolase from *Aspergillus niger*) and the performance evaluation of the ISM when reaching a local minimum [33]. The results showed that applying ISM resulted in successfully escaping from the local minimum.

#### 2.1.3. Combinatorial Active-Site Saturation Test (CAST)

Strategies for evolving properties such as substrate recognition and catalysis specificity, including stereochemistry of reaction and selection of improved enzyme for resolution of racemic mixtures and precise enantio-selectivity, have been proposed over the last 5 years by selecting one or more amino acids in the active site pocket or in its close proximity. The best example of a systematic approach to this end is represented by the Combinatorial Active-site Saturation Test (CAST). In this approach, pairs of amino acid residues pointing towards the active site of an enzyme are chosen for complete randomization. The selection of residues is made on the basis of geometric assumptions that suggest choosing amino acid pairs along the sequence of loops, helices or β-sheets. For example, two residues pointing both to the catalytic pocket will for instance be n and n + 2 along the sequence in a β-sheet and n and n + 4 in a α-helix. The randomization of each pair generates a CAST library with 20^2^, *i.e*., 400 possible variants. The limited size of each CAST library allows an oversampling of 3,000 clones for statistically significant screening coverage, thus drastically reducing time and cost efforts. The results from each CAST library can then be combined pairwise by multiple mutations or by iterative strategies and re-randomized as explained above. The impressive results obtained with enzyme specificity and enantio-selectivity [32,34] highlight the suitability of the method to evolve new functions for biocatalysts. Lipases are a good example of the powerful application of CAST. The results achieved on lipases support the suitability of SSM-based methods for biocatalysts improvement, as lipases certainly constitute the core business in key industrial processes such as detergent additives, food processing and biomass pretreatment, bearing a significant impact on the global biocatalysts market that is expected to reach $7.6 billion by 2015 [35]. The availability of the CASTER software provides a very powerful tool for assigning the residue pairs for randomization on the basis of a crystal structure or a homology model. This makes the approach easy to test with several different enzymes in reproducible conditions. The main group working with this approach is that of Reetz [29], but recent applications from other research groups highlighted equally important results [36]. The limitation of the method, that requires as ideal starting point a substrate-bound crystal structure of the biocatalyst to be targeted, can be overcome in most cases by homology modeling, docking tools and in general by available bio-computing techniques.

#### 2.1.4. B-Factor Iterative Test (B-FIT)

The focus of the B-factor iterative test (B-FIT) is the protein scaffold stability, more so than the detail of the catalytic pocket, and therefore it can guide the improvement of parameters such as thermostability that is known to not necessarily relate to the active site residues. The B-factor, or “temperature-factor”, can be calculated from crystallographic data and indicates the static or dynamic mobility of an atom or groups of atoms. The B-FIT approach therefore relies on the principle of ISM combined with criteria for selecting the crucial sites that are based on the availability of B-factor values, *i.e*., on information about the protein scaffold mobility. The hot spots are identified using software called the B-FITTER. This averages the B-factors available from X-ray crystallographic structures and it relies on the principle that high B-factors are signature of very flexible regions of the protein scaffold. Iterative mutagenesis at these flexible sites of the enzyme aims at increasing their rigidity and therefore improving the thermal stability of the enzymes to be used in industrial processes or bulk applications. The test cases that show the most impressive results to date are once more regarding the lipases, enzymes that need to be thermostable as they are typically added to detergents for mid to high temperature biological activity. The availability of the crystal structure is much more of a prerequisite here and therefore the bottleneck. Another limiting point could be the matching of an increased rigidity for thermostability [37,38] and also for stability against denaturating agents such as organic solvents [39], together with an adequate dynamic range necessary for the structural rearrangements that occur in many enzymes during catalysis. The interesting further proof that the approach is based on a measurable parameter directly correlated with flexibility and thermolability is the re-engineering of the thermostable lipase from *Pseudomonas aeruginosa*. This enzyme maintains catalytic features while dramatically decreasing its thermal stability, with Tm halved from 72 °C to 36 °C. This was achieved by reversing the approach illustrated above, by selecting and randomizing few chosen positions with a lower B-value according to the B-FITTER software to achieve destabilization of the original enzyme [40].

Another recent strategy able to select regions of potential protein flexibility and therefore hot spots to be subjected to saturation mutagenesis for tuning thermostability was named Coevolving-Site Saturation Mutagenesis (CSSM) [41]. The method relies on computational algorithm [42] and sequence alignment to select coevolving residues and/or pairs of co-evolutionary interactions that are then targeted with saturation mutagenesis to generate variants selected for improved thermostability. 

#### 2.1.5. Cassette Mutagenesis and Other Approaches for Multisite Saturation Mutagenesis

**Figure 3:**
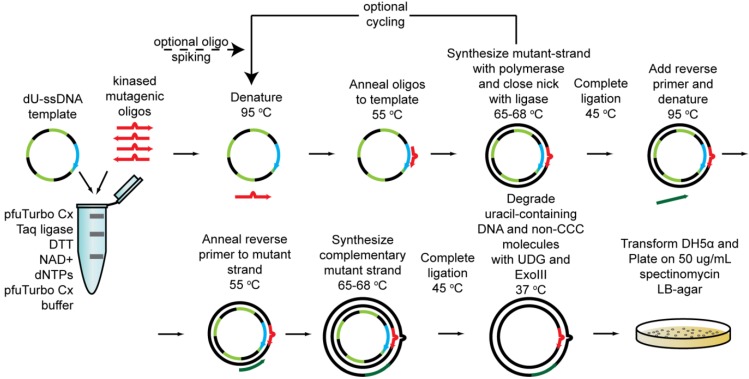
Schematic of PFunkel mutagenesis strategy (adapted from Firnberg *et al*. [44]).

Cassette mutagenesis is one of the classical approaches for systematic mutagenesis at fixed positions [43] that can be chosen for multisite saturation mutagenesis. It is usually applied when a relatively short DNA sequence is to be mutated by synthetic oligonucleotide primers designed to introduce multiple mutations at targeted amino acids in the same stretch of primary sequence. The excision and re-introduction of the mutated cassette by molecular biology techniques, such as introduction of restriction sites and ligation in the original vector, makes it a time consuming procedure. Likewise, methods that follow the classical Kunkel mutagenesis approach using ssDNA also suffer from the same drawbacks. However, a recent novel approach named PFunkel [44] has been proposed that re-interprets the Kunkel methodology and that can be performed in one day in a single test tube (Figure 3). This was applied to create a library with site-saturation at four distal sites and it was tested on TEM-1 β-lactamase gene to produce a library of 18,081 designed variants: library sequencing attested that a 97% coverage of the expected variants were present in the library, and this was then screened for variants resistant to the ß-lactamase inhibitor tazobactam. 

**Figure 4:**
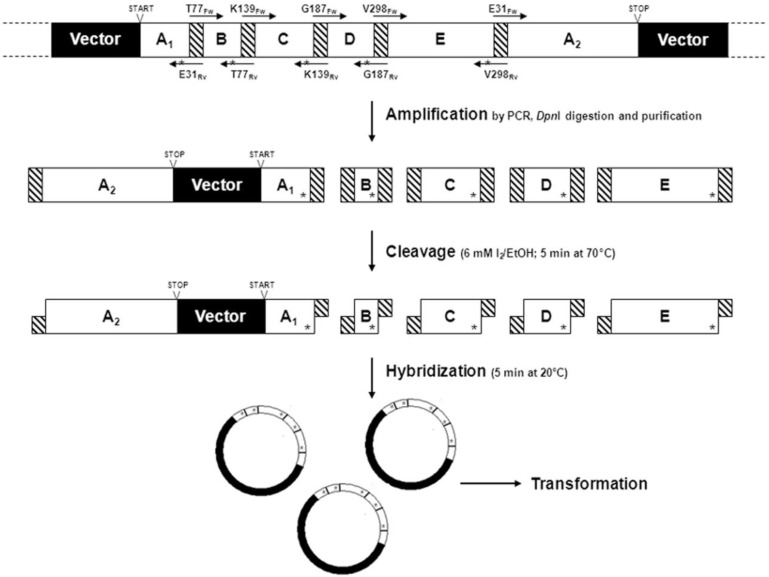
The 4-step strategy for the simultaneous saturation of 5 independent codons by OmniChange (adapted from Dennig *et al*. [45]).

Another recent strategy to simultaneously introduce saturation mutagenesis at multiple sites (up to five codons) was proposed by Schwaneberg and co-workers [45]. The scheme of this approach, named OmniChange is reported below (Figure 4).

#### 2.1.6. Reducing Amino Acid Alphabet

The query for strategies that can reduce library size without limiting functional variants has led to several attempts to restrict the amino acid alphabet. All “reducing amino acid alphabet” approaches aim at defining a small set of a few representative amino acids that can efficiently function as building blocks for all proteins. Saturation mutagenesis performed with a restricted alphabet at multiple sites has the advantage of generating smaller and potentially smarter libraries. The risk is to over-simplify the subset and exclude subtle and specific properties of some amino acids. The design of the subset chosen is therefore a very delicate step. The main efforts in this direction came from Hilvert [46] and Reetz [47] and co-workers who respectively proposed a reduced alphabet of 9 and 12 representative amino acids applied to the design of an enzyme able to function as chorismate mutase [46] and to the engineering of the active site of an epoxide hydrolase [47]. Although the function of these enzymes can efficiently be complied by this simplified catalyst, the stability of the protein was not entirely satisfactory, as an undesired enhanced flexibility was observed in the enzyme designed with the 9 amino acid reduced alphabet [46]. In other cases the reduced amino acid alphabet was specifically designed on the basis of sequence alignment and consensus variants and the strategy applied to the focused mutagenesis of a phenyl acetone monooxygenase [48]. The main advantage of this method is well highlighted by the rigorous comparison of library coverage when randomizing multiple positions with the alternative codon NNK for the 20 amino acids and with the codon NDT (D: adenine/guanine/thymine) encoding for the reduced 12 amino acid alphabet. The number of variants to be screened in the NDT library for 95% coverage is less than 500 for a two position randomized mutant and 5,000 for a three position mutant. In the case of NNK library for a two positions mutant a screening of 3,000 is required, while for a three positions mutant the screening of 10,000 variants only covers 25% [49]. For the purpose of reducing library redundancy, and consequently screening efforts, a more convincing strategy has recently been proposed by designing appropriate mutagenic primers that can cover the 20 amino acids with only 22 codons [50].

### 2.2. Statistical Robustness of the Method and Requirements for Library Screenings

A key point of all laboratory evolution techniques is library screening and variant selection, which is tightly intertwined with the statistical analysis of library coverage. Although SSM is a focused strategy among the wider landscape of directed evolution approaches, the importance of these two aspects is crucial and bears implications for judging SSM and evaluating its potential application. Therefore a brief coverage of the topic will be presented below with a focus on relevance to SSM strategy.

**Figure 5:**
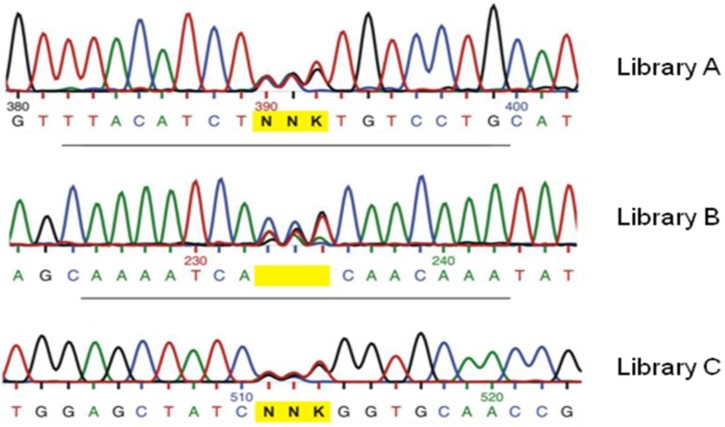
DNA sequencing of the three libraries produced for evaluation of the randomization efficiency on selected position in hydrogenase gene: the targeted position is properly randomized for NNK in library A and C (K either a T or a G), while only partial degeneration is present in library B.

The saturation mutagenesis methods usually aim at the production of relatively small and high quality libraries, whose screening could cover all different variants with an established degree of confidence. It is therefore crucial to acknowledge the importance of statistics [51,52,53,54] for estimating the number of analyses to be performed and determining the sample size to be screened. In most methods, with the exception of recently proposed alphabet reducing [47] and redundancy reducing approaches [50], the distribution of encoded amino acids is impaired in frequency due to the genetic code redundancy. Thus a library constructed with NNN configuration will have leucine represented six times for every tryptophan. As a result, the sample size should always be calculated on the basis of nucleotide rather than amino acid diversity. The statistics of the process is described by the following equation [54].

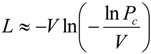
(1)
where V is the number of possible variants (64 for NNN degeneracy, 32 for NNK degeneracy of a single codon), L is the number of clones in the library, Pc is the probability of completeness of the library. Thus, the equation correlates the number of clones in the library with the probability that each clone is actually present in the library at least once. The same holds for the screening. As an example, the screening of 360 clones obtained by a NNK degenerated library at a single site, providing 32 different codon variants, ensures a probability of 99.96% that each variant has been tested at least once, while lowering the screened clones to 247 lowers the probability to 98.59%. The assumption is of course that the NNK or NNN degeneracy and the SSM protocol applied is not affected by biases and that the incorporation of each codon is equally possible. This is not always the case and controls of library completeness can be performed by sequencing the entire library mixture (Figure 5) and/or randomly selecting a few clones (either positive or negative) to demonstrate that a good variability of codons for different amino acids are actually present [55].

The higher the degeneracy of the library, the higher the number of clones to be screened in order to have a significant probability of coverage of all mutants. For example, to achieve 95% (the threshold for significance is usually set to this value) probability to cover all mutants in a 1,024-fold degenerated library, it has been estimated that about 3,000 clones should be screened. Most SSM experiments reported in the literature cover the mutated library between two to four times on a basis of nucleotide diversity (e.g., 64–128 clones are usually screened for a 32-fold degenerated library). Often incomplete screenings of large libraries can allow identification of variants with desirable features [56]. This strategy is, however, prone to the statistical uncertainty of missing clones with remarkable properties. To reduce the library size and overcome genetic code redundancy, mixtures of highly specific primers can be used instead of a degenerated primer. Therefore, 19 primers (one for each specific amino acid alternative to the WT amino acid) can be used to randomize each codon. This can also be applied when a bias in codon incorporation is present (Figure 3 library B) and a properly randomized library cannot be synthesized.

Recent novel techniques and designed primers were proposed to further reduce codon redundancy and to ensure equal probability of coding each amino acid, by limiting the code to 22 triplets covering the 20 standard amino acids [50].

The researcher in the laboratory designs the selection of desired variants by the application of an appropriate screening method. The general rule that “you get what you screen for” indicates that this step is a particularly crucial one and often represents the bottleneck for the success of directed evolution in developing improved or new biocatalysts. The selection method must be rapid, sensitive and allow for the clear identification of the desired properties, implying that the screening must not be marred by undesired selection criteria. 

The fully randomized methods of shuffling or error prone PCR implies the production of very large libraries and therefore the requirement for equally powerful high-throughput screening techniques, such as phage display or other more recent molecular display methods [57,58]. These methods enable the screening of up to 10^12^ protein variants, but usually rely on costly equipment and are only suitable for very focused applications. On the contrary, *in vivo* selection of suitable enzymes by setting experimental parameters so that conditional cell survival is linked to the desired biocatalyst function usually is low cost and allows high-throughput performance. Unfortunately, instances have been reported in literature in which surviving cells bypassed the desired enzyme expression. Also by setting a high threshold there is the probability that low activity variants with potential interest are excluded.

**Figure 6:**
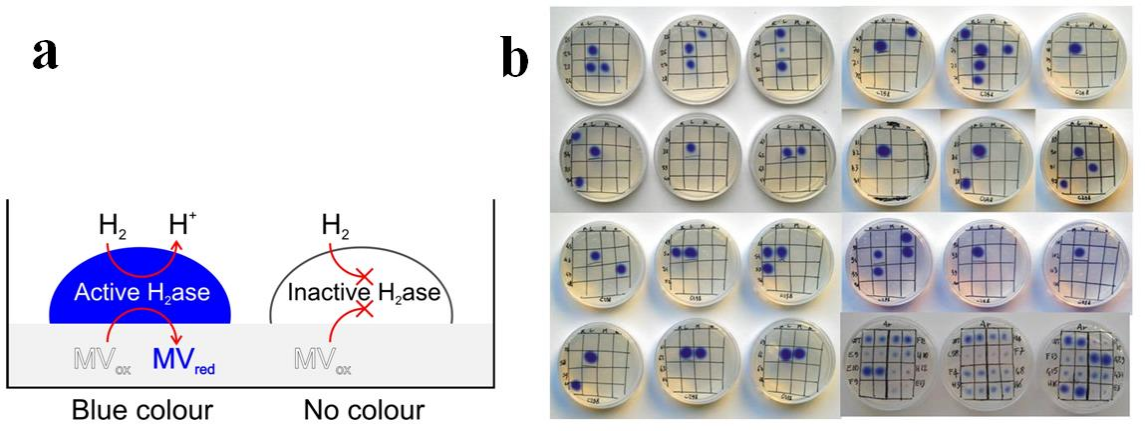
(**a**) Scheme of the principle of on-colonies activity test for a [FeFe] hydrogenase [55]; (**b**) Example of the screening results.

The application of spectrophotometric [59] or fluorimetric [60] platform that can screen for the desired product formation or at least for substrates and co-substrates consumption by the biocatalyst of interest is a more versatile option that can be extended to very specific catalysis, such as stereo-specific production of chiral compounds [61], biodegradation of recalcitrant poly-aromatic hydrocarbons [62], for the synthesis of drug metabolites [63,64] and the turnover of novel chemical entities for drug synthesis, such as 1,2,5-Oxadiazole derivatives [65], for hydrogen evolution and uptake [55,66]. The superior specificity and versatility of such assays is reached at the cost of lowering the through-put efficiency, even for quick assays that can be performed on multi-well plates, directly on cell lysates or colonies (Figure 6) [55]. Compared to fully randomized methods, saturation mutagenesis, which provides small but high quality libraries, allows the application of such focused and function-specific screenings whilst maintaining statistically sound library coverage.

## 3. Recent Successful Applications

An increasing number of recent papers proposes the application of saturation mutagenesis to biocatalysts of applicative interest, for “greener” industrial processes [67,68] improved bulk enzymes [41,69,70], biotechnology [71,72] bioremediation [73], fine chemical synthesis [74,75,76,77,78,79,80], biofuels production [55,81,82] and biomass exploitation [82,83,84,85]. 

A selection of relevant successful examples published in the past 5 years is presented below. The report is divided in two sections: (1) enzyme classes with high impact on industrial processes and fine chemistry (*3.1*) and (2) enzyme classes with applications in environmental care and production of clean energy (*3.2*). Some classes, for example oxygenases, are relevant to both and therefore are listed twice. 

### 3.1. Enzymes Relevant for Industry

#### 3.1.1. Lipases

**Figure 7:**
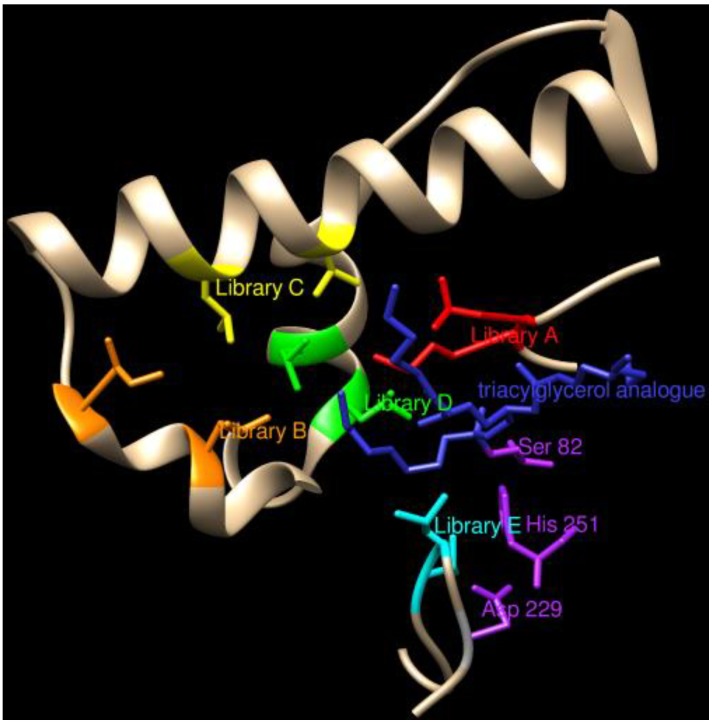
Scheme of the structure of *P. aeruginosa* lipase active site pocket (PDB: 1EX9) with the targeted sites (library A: Met16/Leu17 in red; library B: Leu118/Ile 121 in orange; library C: Leu131/Val 135 in yellow; library D: Leu159/Leu162 in green; and library E: Leu231/Val 232 in cyan). Ser82, Asp229, and His251 (in violet) represent the catalytic triad. A substrate analogue (RC-(RP,SP)-1,2-dioctylcarbamoyl-glycero-3-O-octylphosphonate) covalently bound to Ser 82 is shown in blue.

Lipases are considered as benchmark enzymes for biocatalysis: Lipolase®Ultra and LipoPrime® are the first examples of engineered lipases for commercial distribution in detergent industry. They are also exploited in other industrial large-scale processes and as dedicated catalysts for highly stereo-specific catalysis in fine chemistry. Saturation mutagenesis has played a key role in engineering several lipases both for thermal stability and enantio-selectivity, with at least 20 research papers published in the last 5 years. Among groups involved in lipases engineering, Reetz and co-workers achieved relevant results [29,32,34] by applying SSM, ISM and CAST for enhancing enantio-selectivity and B-FIT for tuning thermal stability properties. The SSM approach was applied to *Pseudomonas aeruginosa* lipase, a well-known catalyst applied to hydrolysis of carboxylic acid esters and transesterification of primary and secondary alcohols, with the aim of redesigning the substrate recognition pocket to enable catalysis on more bulky substrates, such as benzoic acid esters. Ser 82, the key residue for the stabilization of the oxyanion intermediate, was not addressed by the mutagenesis since it structurally belongs to a more distant portion of the enzyme, while the CAST strategy guided the selection of five pairs of residues pointing towards the active site and defining the recognition determinants of the hydrophobic portion of the ester. Five libraries were produced by simultaneous saturation mutagenesis at the two defined positions, that is library A to E: Met16/Leu17, Leu118/Ile 121, Leu131/Val 135, Leu159/Leu162, and Leu231/Val 232 (Figure 7). 

The five libraries of 3,000 variants each were then screened with a spectrophotometric method by testing 11 different substrates. The total reactions performed (165,000) allowed to select eight hits from libraries A and D, consistent with the focus on hot spots even within the restricted region analyzed. Although the success rate in this case was lower than for other SSM reported approaches, the few selected variants showed an impressive gain in function, for instance by binding adamantyl carboxylic acid esters that are not recognized by the WT, as well as showing a 100 fold increase in the rate of hydrolytic activity on substrates that are poorly recognized by the WT [29]. Further works on the same enzyme by ISM highlighted the enormous potentiality of iterative saturation versus other methods such as error prone PCR, shuffling and even the previous SSM, in particular for enhancing the stereospecificity of reactions. In fact, a more recent paper reports, on the same enzyme, the gain of function for the bulky 2-phenylalkanoic acid esters that are not recognized by the WT and the selection of variants with enantio-selectivity of E = 436, achieved with only small mutant libraries and thus a minimum of screening effort [34].

Also, the selection of a *Pseudomonas aeruginosa* lipase engineered variant with an enatioselectivity of E = 594 for the kinetic resolution of a chiral ester from an ISM library upon screening only 10,000 transformants is an unprecedented result [32], given that by directed evolution based on DNA shuffling, only a best variant value of E = 51 (ee > 95% at 24% conversion) could be obtained by screening about 50,000 transformants [86]. In the specific case of variant 1B2, characterized by a high E value of 594, this was produced by ISM starting from three libraries with simultaneous randomization at two near sites each, namely library A (Met16/Leu17), B (Leu159/Leu162), and C (Leu231/Val232). The selection of a best hit from library B with E = 8 (Leu162Asn) was followed by a second round of randomization on library A with DNT codon that simplifies amino acid alphabet by excluding Leu and therefore back-mutating to the original amino acid Leu17. This led to the highly optimized 1B2 variant (Met16Ala/Leu17Phe/Leu162Asn). 

A very recent paper [70] reports the application of ISM and CAST to the engineering of *Candida antarctica* lipase B (CALB; Novozyme 435), a top industrial biocatalyst applied in kinetic resolution of racemic alcohols and amines, desymmetrization of diols and in other stereoselective synthesis of chiral intermediates for pharmaceuticals, polymer chemistry, and protection/deprotection technology. CAST guided selection of active site residues and ISM cycles with restricted alphabet using NDT degeneracy allowed for the isolation of two best mutants that were tested on several substrates for enhancement of activity and S- or R-stereospecificity. These two best hits, named RG401 and SG303 were tested on four representative chiral α-substituted carboxylic acid esters. Specificity constants k_cat_/K_M_ from 13 to 270 fold higher than WT were achieved for SG303 with E (S) up to 64. The other mutant, RG401, acquired an enantio-specificity with E (R) up to 68 although the specificity constants were only slightly higher or of the same order of magnitude of WT. On *Candida antarctica* lipase A (CALA), Bäckvall and co-workers [36] applied the CAST strategy to enhance the performance of the catalyst by building two reduced libraries based on the NDT degeneracy: library FI (Phe149 and Ile150) with side chains directed toward the R-methyl group of the substrate and library FG (Phe233 and Gly237) with side chains defining the acyl-recognizing pocket of the active site. The reduced library size allowed a high coverage (>95%) by screening only 600 variants per library and allowed to select variants with E values of 45–276 (WT E value is only up to 20) and up to 30 fold increased activity for seven different esters used for the preparation of enantiomerically pure 2-arylpropionic acids, important building blocks for the synthesis of non-steroidal anti-inflammatory drugs such as Naproxen, Ibuprofen, and Flurbiprofen. The same group recently reported a further enhancement where CALA variants with high activity and E value of 100 towards an ester of ibuprofen were obtained. This substrate had failed to be recognized efficiently and with high stereospecificity by variants selected previously [80]. 

The robustness of the saturation mutagenesis methods, in particular with the B-FIT strategy, for thermal stabilization and destabilization of lipases for catalysis at desired optimal temperature, has already been discussed (Section 2.1.4) [37,41] and the same approach has proven to be suitable for stabilization towards other denaturing agents such as organic solvents [39]. 

#### 3.1.2. Esterases and Other Hydrolases

Esterases are also extensively used in biocatalysis: saturation mutagenesis strategies have been applied to some enzymes of this class, in particular for the esterase from *Pseudomonas fluorescens*. Enhancement of enantio-selectivity [87] of this enzyme was pursued by the use of simultaneous saturation mutagenesis at four hot spots, with restricted alphabets chosen on the basis of more frequently represented amino acids in structurally equivalent positions on the basis of 1,750 known sequences. This approach granted variants with improved rates (up to 240-fold) and enantioselectivities (up to E(true) = 80) towards 3-phenylbutyric acid esters with the advantage of a relatively limited effort for screening these “small but smart” libraries. As for thermal stabilization, the same enzyme was targeted at three sites, selected by B-FIT strategies, granting an enhanced stability of almost 10 °C higher than the starting catalyst [88].

Other class 3 hydrolytic enzymes that were targeted by saturation mutagenesis for improved catalysts development include epoxide hydrolases, already mentioned as test cases for the development of focused restricted alphabet libraries [46]. A limonene epoxide hydrolase from *Rhodococcus erythropolis*, performing a rare one-step mechanism, was also targeted by ISM to select variants with high stereoselectivity on substrates different from the natural limonene epoxide. Active site binding pocket residues were selected and the codons randomized with a reduced amino acid alphabet strategy. Variants obtained from 5,000 screened hits can catalyze the desymmetrization of cyclopentene-oxide with stereoselective production of (*R*,*R*)- or (*S*,*S*)-enantiomers, the desymmetrization of other meso-epoxides and kinetic resolution of racemic substrates [89].

Because of its potential usefulness in β-lactam antibiotics synthesis, α-amino acid ester hydrolases were also chosen for improvement by saturation mutagenesis. A study was performed on 13 residues not directly involved in substrate recognition (based on the crystal structure of a protein-cefprozil complex) that were individually randomized in the enzyme from *Xanthomonas rubrillineans.* Mutants were selected with improved synthetic activity of *p*-hydroxylcephalosporins with a 23%, 17% and 64% increase in product yield for cefadroxil, cefprozil and cefatrizine, respectively [90].

Another biocatalyst relevant for bulk applications and belonging to the hydrolase class is represented by phytase, commercialized as an additive to poultry and swine feeding preparation in order to enhance digestibility of phytate and increase phosphorus assimilation. The challenge for enzyme engineering here is to enhance the stability of the catalyst not only to temperature but also to gastric degradation and to very low pH environment of the digestive tract so that the enzyme can still be active during the feeding process. Industry interest in this biocatalyst and in mutagenesis approaches aiming at improving its performance is testified by a paper dating back to 2004 [91] published on a research carried out by the company Diversa Corporation, San Diego, CA, USA. The dhlA phytase encoding gene from *Rhodococcus* was chosen to apply saturation mutagenesis with NNK codon systematically to all 431 positions of the protein sequence and screening was performed on at least 150 clones for each individually produced library. By isolating the best single mutants for enhanced low pH stability after heat treatment of the variants, therefore combining a selection for two desired properties, the authors selected 14 single mutants with improved properties and performed a combinatorial strategy and a second screening to isolate synergic and additive effects of multiple mutations. Variant Phy9X, with eight combined mutations, led to a novel biocatalyst with the ability to reversibly renature upon heat treatment and also function at process temperatures of 65 °C, with specific activity at the same level of WT but extending to below pH 2.5 and a 3.5 fold enhanced stability to gastric degradation.

#### 3.1.3. Oxygenases and Other Redox Enzymes

Among redox enzymes, oxygenases have been key examples of the possible improvements brought by protein engineering to the efficiency of enzymes, and particularly of biocatalysts: the focus on cytochromes P450 and Baeyer-Villiger monooxygenases has always been maintained when proposing rational, semi-rational and randomized techniques of laboratory evolution with the seeding work of the groups of Arnold and Reetz, respectively and of many other groups that proposed directed evolution of these versatile biocatalysts. More recently, in particular for P450s, an increasing number of papers proposed saturation mutagenesis alone, or in combination with random techniques, to refine particular applications supported by this class of enzymes in fine chemical synthesis. This also extends to other non-heme iron oxygenases used for enantioselective synthesis of pharmaceutical compounds and chiral sulfoxides [74].

**Figure 8:**
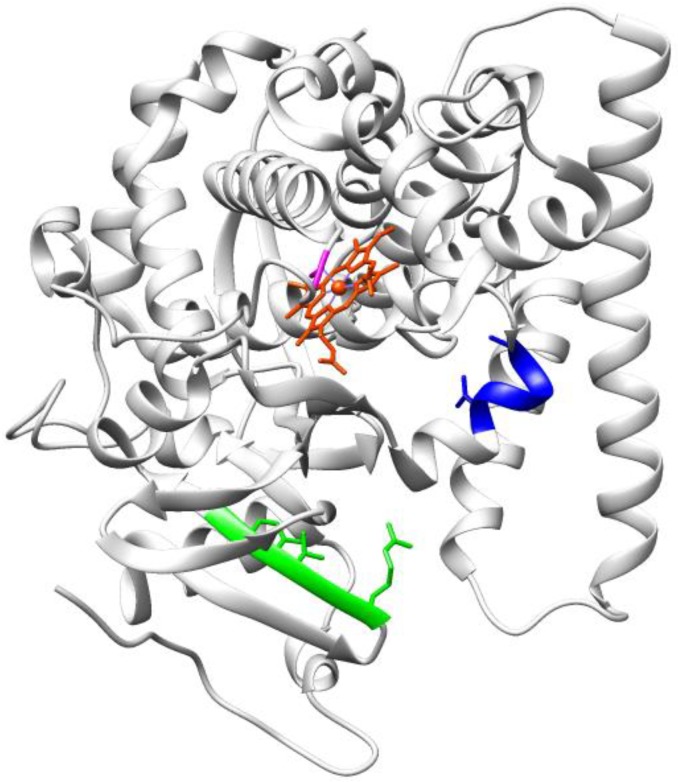
Structure of P450 BM3 heme domain (PDB: 2HPD) showing the target sites A (Arg47, Thr49, Tyr51) in green and B (Val78, Ala82) in blue. Heme is shown in red, the Fe coordinating Cys 400 is in magenta.

Starting from P450s, saturation mutagenesis seems to be the preferred method to enable enhancement of both regio- and stereo-selectivity for the C-H hydroxylation reactions that are of interest in fine chemistry. Steroid hydroxylation by cytochromes P450 in controlled positions leading to enantiomerically pure products is one of the most targeted goals of industry. The results achieved with saturation mutagenesis in the last few years benefit from the knowledge in terms of key spots relevant to improving enzyme performance acquired through directed evolution. Further specific improvements have been made possible by saturation mutagenesis. A very recent work by Glieder and co-workers [75] addressed the two active site residues 216 and 483 by saturation mutagenesis to generate all 400 possible combinations of amino acids. A double mutant of WT CYP2D6 resulted in a high regio-selectivity for hydroxylation at the 2β-position, instead of the 6β-position, suggesting that the mutation F483G could be preferential to the reported F483I for regio-selectivity in the well-known protein hot spot F483. Moreover, a previously obtained mutant F87A of P450 BM3, was further targeted by ISM for selective hydroxylation of testosterone in either of the two possible products 2β- and 15β-alcohols [92]. The CAST approach was applied to choose appropriate sites surrounding the binding pocket. The 20 residues selected as possible candidates for ISM were grouped into nine sites of neighboring amino acids, as this is known to maximize the cooperativity more than the additive effects and it is obviously useful to reduce the library size. Site A (Arg47, Thr49, Tyr51), and site B (Val78, Ala82) were targeted (Figure 8) first with NDC codon degeneracy at the three spots of site A with the need to screen only 430 transformants for a 95% coverage. 

The two-residues at site B were randomized using NNK codon degeneracy. From this first screening, highly 2β-selective mutants (97%) were obtained from library A while 15β-selective variants, also reaching 91% regio-selectivity on testosterone, were found mainly in library B. The best variant from library B was then subjected to randomization at site A with some variants reaching 96% regio-selectivity on testosterone (R47Y/T49F/V78L/A82M/F87A) while a variant from library B only selected on testosterone (V78V/A82N/F87A) was able to reach a 100% regio-selectivity on other steroidal substrates such as progesterone. Moreover, some mutated variants displayed increased coupling of product formation with NADPH consumption. This ISM approach was also characterized by a limited amount of screening, the step that is normally considered the bottleneck of directed evolution. 

A refinement of previously evolved mutants of P450 BM3 was also proposed in 2008 for production of indigo and indirubin by indole hydroxylation [93]. Starting from a variant A74G/F87V/L188Q obtained by random methods and directed evolution, and by applying saturation mutagenesis as a refinement of catalyst properties, granted two variants with increased catalytic efficiency up to six times that of the starting variant, with improved regio-selectivity for 3-hydroxyindole, leading to 93% indigo production *vs.* the initial 72%. One of the variants also showed increased coupling efficiency with NADH. The overall result nicely supports the importance of synergy of random and saturation mutagenesis approaches for optimized catalysts production.

Recently, another study has been published [94] on P450sca-2 from *Streptomyces carbophilus* to be employed in the synthesis of the cholesterol-lowering drug pravastatin. Here the saturation mutagenesis was applied to enhance electron transfer efficiency in a hybrid P450sca-2/Pdx/Pdr functional system by targeting residues at the interface between the electron transfer moiety putidaredoxin (Pdx) and the catalytic P450sca-2. Three rounds of ISM granted a variant with a 10 fold improved catalytic performance. 

The other important enzymes belonging to the oxygenase class and successfully targeted for improvement by saturation mutagenesis [95,96] are represented by the Baeyer-Villiger monoxygenases (BVMO), able to perform specific reactions on racemic mixture of various ketones to obtain enantiopure lactones, conversion of prochiral ketones in chiral lactones and oxidation of organic sulfides. Although novel Baeyer-Villiger monoxygenases with tuned substrate specificity can be found in diverse microbial populations [97,98,99], there is the need to evolve BVMOs with specific performance in biosynthesis. This can be done with random or SSM laboratory techniques.

**Figure 9:**
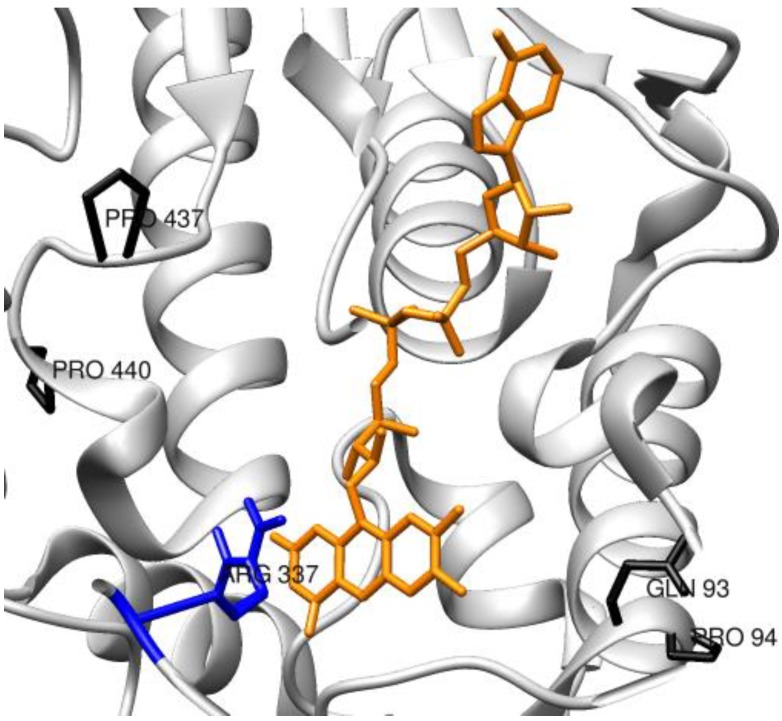
Scheme of the active site of PAMO (PDB: 1W4X) with targeted residues Pro440, Pro437, Gln93 and Pro94 (in black). FAD is shown in orange; Arg 337, involved in catalysis, is shown in blue.

A thermostable phenylacetone monoxygenase (PAMO) belonging to the BVMO group was successfully engineered by saturation mutagenesis to perform catalysis on 2-aryl, 2-alkylcyclohexanones and a bicyclic ketone that are not recognized as substrates by the WT enzyme [96]. Given that a CAST approach previously applied to positions 441–444 belonging to a loop next to the binding pocket, were only partially successful [48], only positions 440 and 437 were targeted instead, where the first amino acid is located in the second sphere, and therefore not in direct contact with the substrate (Figure 9). Pro440 was identified to play a key role, since several mutants generated at this position granted an enhanced percentage of conversions and improved enantio-selectivity for substrates not recognized by the WT. Since in this case the library was apparently not covering the entire range of variants at position 440, the missing variants Pro440Tyr and Pro440Trp were produced by site-specific mutagenesis, with the aim of exploring the entire range of amino acid properties at this position for the enhancement of the biocatalyst performance. Further work on the same enzyme [95] targeted positions 93 and 94, located in site distal from the binding pocket chosen on the basis of the crystal structure, with a simultaneous saturation mutagenesis using a NDT codon to reduce degeneracy. A double mutant Gln93Asn/Pro94Asp was selected for its acquired activity on an otherwise inert 2-substituted cyclohexanone derivatives and it was found to be able to catalyze the conversion to the corresponding lactones with high enantio-selectivity. These results have been rationalized by a rearrangement of the H-bonds and salt-bridge networks in the protein, much alike an induced allosteric effect.

In order to enhance the performance of biocatalysts for fine chemistry, for example, for the synthesis of chiral sulfoxides and asymmetric ketone reduction, other redox enzymes such as nitrobenzene dioxygenase [77], alcohol dehydrogenase [78] and carbonyl reductase [79] were also recently optimized by saturation mutagenesis.

An interesting example of active site saturation mutagenesis recently published, targeted an unusual non-heme iron dioxygenase, belonging to the class of α-ketoglutarate dependent dioxygenase [74]. This enzyme is involved in the biosynthesis of carbapenem-3-carboxylic acid, the core building block of the all carbapenems, including Meropenem and Imipenem. This is a relatively new class of β-lactam antibiotics of great importance as therapeutic agents given the increasing bacterial resistance to an older class of antibiotics. In order to dissect and better understand the molecular determinants of the biocatalyst that promote the epimerization and desaturation crucial for the biosynthesis of the core of cabapenem, SSM was applied to six active sites and four second sphere residues of the dioxygenase, generating point as well as double mutant libraries. The importance of Tyr 67 for catalyst engineering was highlighted together with the advantage of promoting a two step reaction mechanism, including epimerization and desaturation, with release and rebound of the intermediate to ensure complete desaturation and avoid the frequent aborted cycles that are observed in the native enzyme due to a difficult rotation of the intermediate required in the catalytic pocket in the full reaction.

Other redox enzymes optimized by SSM include dehydrogenases as the previously cited alcohol dehydrogenase from *Thermoethanolicus brockii* [78] and the meso-diaminopimelate dehydrogenase from *Symbiobacterium thermophilum* [100] successfully exploited for the synthesis of D-phenylalanine, thanks to a 35-fold increase in specific activity of the variant compared to the WT.

### 3.2. Enzymes Relevant to Environmental and Clean Energy Approaches

The use of enzymes in environmental applications include biocatalysts able to detoxify pesticides such as atrazine, chlorinated polyaromatic hydrocarbons, DDT, toxic compounds in industrial wastes such as phenols, organic solvents, aniline, drugs, explosives and chemicals resulting from military operations, among which trinitrotoluene (TNT) and G-series organophosphorus toxins contained in nerve agents like Sarin and Cyclosarin. These are usually xenobiotics particularly recalcitrant to degradation by bacteria and fungi, given that their natural enzymes, though powerful catalysts for bioremediation [101], have not evolved under the selective pressure of such compounds, as these organisms were not massively exposed to these compounds until very recently. In this respect, protein engineering by laboratory-driven evolution is of unique importance for what it can deliver. Several important results have been achieved in this respect by random directed evolution approaches both on P450 enzymes acting on pollutants [1,4,62] and on hydrolytic enzymes, for example on paraoxonases (PON) for detoxification of organophosphorus toxins [102], but an increasing number of works have recently tackled the same problem by applying SSM methods.

SSM relevance for improvement of lipase applications as an industrial catalyst has already been discussed in Section 3.1. Lipases are also relevant for clean energy issues in the transesterification of triacylglyerol with methanol for biodiesel production [81,103]. These have many advantages over traditional base or acid catalyzed approaches, but natural lipases often lack the required stability and efficiency in the high methanol concentrations used for biodiesel synthesis, limiting their practical use. Directed evolution techniques were very recently applied to the lipase from *Proteus mirabilis* to enhance methanol tolerance and allow its industrial application as a biocatalyst. The dieselzyme variant 4, evolved by randomized methods (error prone PCR) and site-directed mutagenesis to combine beneficial mutations, shows a 30-fold increase in the half-inactivation time to temperature (50 °C) and a 50-fold longer half-inactivation time in 50% aqueous methanol [81]. Although saturation mutagenesis was not the chosen technique for this approach, the authors foresee the application of CAST and structure guided ISM for further refinement of the obtained catalyst. 

Enhancement of performance of enzymes such as cellulases and ligninases, present in nature in a restricted number of organisms, is of high relevance to the production of clean and sustainable energy from renewable sources. These enzymes offer precious tools for waste and poor-value biomass recycling, acting both on recovery of resources for energy production and on management of wastes [104].

The frontier of environmental care and clean energy production is the setup of hybrid systems based on biocatalysts, often interfaced with semiconductor materials [105,106] with the ability to mimic nature in efficient solar energy harvesting and energy storage in transportable fuels of low impact to the delicate equilibrium of our planet. In this respect, photosystems, light activated proteins, CO_2_ fixing enzymes and biocatalysts able to produce fuels such as biohydrogen, bioethanol and biodiesel, are the ideal target of engineering approaches. Many clean-energy production related enzymes (in particular photosystems and hydrogenases) are generally difficult to purify, manipulate and engineer, and therefore the laboratory evolution approaches are still at their first steps of development, but it is foreseen that increasing interest will be devoted to engineering, particularly with SSM methods applied to hydrogenases, nitrogenases, formate-dehydrogenase. 

Here a choice of examples, grouped as in Section 3.1 by enzyme classes or subclasses, focus on the three aspects: detoxification, biomass degradation and clean energy production.

#### 3.2.1. Oxygenases and Other Oxidoreductases for Bioremediation

Oxygenases and more in general redox enzymes represent a class of biocatalysts spanning from P450s to non-heme iron mono- and di-oxygenases and flavoenzymes widely used for the oxidation of toxic compounds. The addition of one or two hydroxyls to a poorly reactive C-H bond, for example in aromatic and aliphatic hydrocarbons, is usually crucial for the initiation of the detoxification and clearance process. The increasing amount of pollutants with halogenated substitutions in aromatic rings, for example in pesticides, and the presence of compounds recalcitrant to biodegradation, poses difficult challenges to protein engineers. SSM techniques are often the selected method to test and modify redox enzymes to recognize a broader substrate range and to attack xenobiotics with a sustainable approach, recovering carbon sources for safe microorganism growth. The catabolic pathways that enable many microorganisms to degrade large classes of aromatic pollutants, often relay on non-heme iron dioxygenases and monoxygenases. These include di-iron oxo-bridged monoxygenases such as methane-monoxygenase, phenol hydroxylase, toluene 4-monoxygenase and toluene-o-xylene monoxygenase. The last two enzymes have been target of early applications of SSM [107,108], as well as refinement of previous successful directed evolution approaches [109]. Further work, more focused on developing enzyme catalysts for bioremediation, has been developed on dioxygenases containing a single iron atom such as ring-cleaving dioxygenases acting on polychlorinated biphenyls [110], aniline [111,112], dinitrotoluene [113] and chlorinated catechols [114]. The engineering of the extradiol dioxygenase (DoxG) that displays a low activity in 3,4-dihydroxybiphenyls ring cleavage was achieved by a combination of error-prone PCR, SSM at hot spots and DNA shuffling applied in sequence. Four residues located within 14 Å of the enzyme active site iron, highlighted by error prone PCR to be relevant for enzyme activity on the screening substrate, were targeted by saturation mutagenesis applied in pairs, grouping Ile-154 and Leu242, Leu-190 and Ser-191. The two resulting libraries were screened with coverage of 99.9% of the possible diversity resulting in variants with 2–10-fold increases in 3,4-dihydroxybiphenyl cleavage rates. After DNA shuffling, a further improvement generated a variant with a k_cat_/K_M_ towards 3,4-dihydroxybiphenyl increased by 770 fold when compared to WT, confirming the feasibility and advantage of a coupled random and saturation mutagenesis approach in biocatalysts activity enhancement. SSM was also applied to an aniline dioxygenase isolated from *Acinetobacter* sp. strain YAA [112]. Substrate-binding pocket residues were selected and the V205A mutation that is possibly responsible for enlarging the binding pocket, was highlighted to lead the oxidation of 2-isopropylaniline, a substrate not recognized by the WT enzyme. The same mutants also shift the substrate specificity from 2,4-dimethylaniline, a good substrate for WT, to 2-isopropylaniline. Another variant, I248L, improved activity towards aniline and 2,4-dimethylaniline by approximately 2 fold. Both residues I248 and V205 were not previously reported to influence substrate recognition, therefore the finding also granted basic information on the enzyme active site determinants for substrate specificity. A further refinement by random mutagenesis on mutant V205A generated variant 3-R21, with improvement in activity towards the carcinogenic 2,4-dimethylaniline of 3.5 fold and retaining WT activity levels towards the natural substrate aniline. Therefore it can be concluded that the laboratory evolution of this biocatalyst generated a powerful tool to detoxify highly hazardous compounds. Another pollutant that has received attention in view of bioremediation strategies is 2,4,6-Trinitrotoluene (TNT), the most common explosive found in past and present war sites, and the intermediates of its synthesis 2,6-dinitrotoluene (2,6-DNT) and 2,4-dinitrotoluene (2,4-DNT) found as soil and water contaminants at TNT production facilities. 2,4-DNT dioxygenase of *Burkholderia* sp. strain DNT can catalyze the oxidation of 2,4-DNT to form 4-methyl-5-nitrocatechol and nitrite, but it has poor activity on other DNTs and nitrotoluens. By applying saturation mutagenesis at position I204 of the catalytic subunit and selecting for nitro-catechol producing mutants (signature of activity on the screened substrate), variants I204L and I204Y were identified [113]. These showed unprecedented activity on 2,3-DNT and 2,5-DNT and 2 to 8 fold improved activity towards 2,4-DNT, 2,6-DNT, 2NT and 4NT. The activity reported on 2,5-DNT, never observed for an enzyme, confirms that new biocatalysts unexplored by natural evolution can be generated by laboratory-driven evolution.

**Figure 10:**
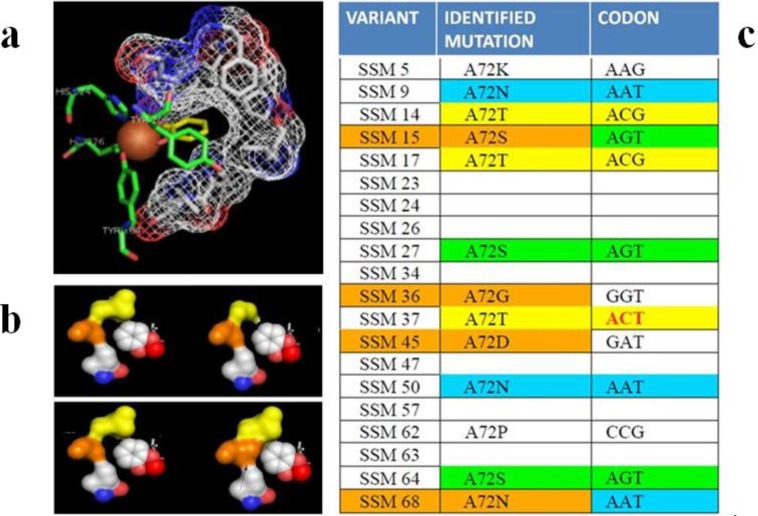
(**a**) Structure of active site of catechol 1,2 dioxygenase highlighting the residue that define the active site pocket (PDB file from crystallographic structure in [117]); (**b**) The effect of reshaping by mutagenesis and SSM on model of substrate/pocket interaction; (**c**) The list of identified and characterized mutants for SSM on position 72 are reported in the table (related to studies published in [114]).

A gain of function on unnatural substrates and an inversion of specificity were also achieved by site directed and site-saturation mutagenesis on a catechol 1,2 dioxygenase from *Acinetobacter radioresistens* S13 [114]. Catechols are the converging metabolites of several aromatic degrading pathways, although natural enzymes usually cannot efficiently oxidize highly chlorinated or variously substituted catechols originated from chloroaromatic, biphenil and nitroaromatic compounds. The advantage of catechol dioxygenases is that these enzymes do not require any supply of reducing equivalent to perform the dihydroxylation and ring-cleavage of substrates, and therefore have a simpler architecture, higher stability and no need for expensive cofactors such as NAD(P)H to perform catalysis. Encapsulated and immobilized forms are also available [115], making them ideal biocatalysts. Mutagenesis on the active site was performed on residues L69 and A72 with a combined site-specific and SSM approach. This led to a series of variants with improved activity on the rarely recognized substrate 4,5-dichlorocatechol (by 2 fold in variant A72S), inversion of specificity for 4-chlorocatechol instead of catechol (variants L69A and L69A-A72G) and gain-of-function for recognition and catalysis on 4-*tert*-butyl catechol, a contaminant of cosmetics and foodstuff banned by EU since it can give sensitizations in patch testing at low concentrations (1%). The effect of active site re-shaping of the chosen mutational sites is shown in Figure 10, together with an example of the SSM obtained variants. An influence on the oxygen binding properties of mutants *vs.* WT was recently highlighted [116] and further work is ongoing in our labs for SSM at other catalytic pocket sites and for production of multiple site variants.

Cytochrome P450 enzymes were also targeted by directed evolution for enhancing the degradation of recalcitrant aromatic and aliphatic pollutants. A recent work by Arnold and co-workers [118] compares combinatorial SSM strategies to the results obtained by random directed evolution. Although in this case it seems that the notable achievements obtained by error prone PCR and several round of random mutagenesis cannot be fully matched by SSM, the paper reports an improved activity on propane and ethane hydroxylation. In this case, nonetheless, a simpler approach by two rounds of error-prone PCR and back-crossing with parental DNA devised in our group on the same P450 BM3, led to variants that are active on highly recalcitrant polyaromatic hydrocarbon (PAH) pollutants, more relevant for environmental concerns, such as chrysene and pyrene [62]. 

A detoxification activity specifically improved by SSM on lactaldehyde oxidoreductase [72] is of relevance for detoxification of furfural, a toxic compound that originates from pre-treatment of cellulosic material. In this perspective the optimized catalyst obtained by SSM, a L7F mutant with a 10-fold higher activity than WT, is crucial both for lowering a toxic compound in an environment and for direct application in cell factory systems to enable cells to improve growth on treated lignocellulosic material. In the cited paper the variant obtained by SSM was also tested for performance in *E. coli* cells and showed a 2-fold higher rate of furfural metabolism during fermentation.

#### 3.2.2. Cellulases, Haloalkane Dehalogenase and Other Hydrolases for Waste Degradation

Hydrolytic enzymes such as cellulases, endoglucanases, xylosidases and β-glycosidases, are increasingly being applied in lignocellulosic waste pre-treatment in combination or in alternative to steam-explosion and chemical treatments for enhanced saccharification of the biomass and lowered environmental impact. The SSM approach to enhance applicability of this class of biocatalysts has been focusing on improvement of thermal stability by the same research group both for an endoglucanase [84] and more recently on a β-glycosidase [85]. In the first case the endoglucanase CelA from *Clostridium thermocellum* was chosen for SSM at protein surface position Ser329. All the variants with improved thermal stability (approximately 5-fold increase in half-life of inactivation) and maintaining hydrolytic activity at WT levels, showed the presence of the S329G mutation. This finding suggested a systematic analysis of other possible substitutions to Gly of surface Ser residues, in line with reported works that Ser to Gly mutations on protein surface may improve thermostability. Thr and His surface residues were also selected on the basis that His and Thr, along with Ser, are generally substituted by Gly on the surface of proteins with enhanced thermal stability compared to their thermo-sensitive homologous. Few residues were also tested for substitution to Pro. A final variant S329G/S269P/H194G, generated by a combination of SSM and site-directed mutagenesis resulted in a 10-fold increase in half-life of inactivation at 86 °C.

A more recent paper from the same group [85] reports a consensus-based semi rational approach that benefits from the results of the previous work to enhance thermal stability of a β-glycosidase BglY from *Thermus thermophilus*.

An SSM approach applied to β-D-Xylosidase/α-L-arabinofuranosidase from *Selenomonas ruminantium* to residue W145 was instead focused on modulating the inhibitory effect of glucose and xylose on this enzyme for application to the saccharification of lignocellulosic waste biomass for biofuels production and as microbial substrate for other biotechnological processes [119]. While the β-D-Xylosidase/α-L-arabinofuranosidase can promote the hydrolytic cleavage of 1,4-β-D-xylooligosaccharides to D-xylose, the high affinity for the product D-xylose as well as for D-glucose hinders its excellent performance as a catalyst. Three variants isolated by screening the SSM library, W145F, W145L, and W145Y, showed decreased inhibition by the monosaccharides and increased catalytic activities up to 70% greater than that of the WT enzyme. 

Another hydrolase applied to a different perspective of waste recycling is represented by a haloalkane dehalogenase DhaA from *Rhodococcus rhodochrous*. This enzyme is able to convert 1,2,3-trichloropropane (TCP) into (*R*)- or (*S*)-2,3-dichloropropan-1-ol, which can be converted into optically active epichlorohydrins, industrially important building blocks for the synthesis of fine chemicals. Enatioselectivity of the WT DhaA was further improved [120] by a pair-wise SSM approach applied to 16 active-site residues not directly involved in the catalytic reaction. A further refinement was then applied to the best R- and S-enantioselective variants by site directed mutagenesis including residues that are not part of the active site. A multi-site mutagenesis protocol with restricted codon usage allowed to finalize two variants, r5-90R and r5-97S with 13 and 17 mutations, that generate (*R*)-epichlorohydrin with 90% ee and (*S*)-epichlorohydrin with 97% ee, respectively.

#### 3.2.3. Hydrogenases and Other Enzymes Relevant to Clean Energy Production

The energy issue has driven a great interest towards hydrogenases as powerful and efficient catalysts for hydrogen production in cell factory systems and in biohybrid fuel cells or solar harvesting devices as catalysts instead of platinum or other expensive rare-metal based materials [105]. Among the three classes of reported hydrogenases, [FeFe] hydrogenases are in this respect the most efficient catalysts due to their high turnover numbers, reaching turnover frequencies up to 10^4^ s^−1^ [121] with a bias toward hydrogen production, but with relatively low overpotential needed as a driving force for catalysis in either direction. Interestingly, perspectives are also discussed in literature on [NiFe] hydrogenases for application in molecular hydrogen conversion for biofuel cells and in NAD(P)^+^ cofactor regeneration.

**Figure 11:**
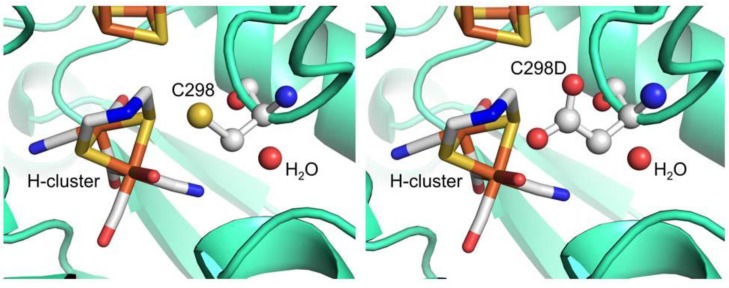
Model of CaHydA structure illustrating C298 (left) and replacement at 298 position with aspartic acid (right) (adapted from Morra *et al*. [55]).

Some limiting features, such as oxygen sensitivity, and the interest to further investigate the complex mechanism of the catalytic center, are increasingly pointing towards the application of saturation mutagenesis techniques to refine hydrogenases for desired applications. Although until now not many papers have been published on this topic [55,122] and in general on mutagenesis and laboratory evolution of all classes of hydrogenases [66,123,124,125,126,127,128], a very recent review from a leading group in the field of [FeFe] hydrogenase foresees imminent development in this direction [129]. In our group we applied saturation mutagenesis to a key residue in the active site of [FeFe] hydrogenase from *C. acetobutylicum* (CaHydA) recombinantly expressed in *E. coli.* This residue, namely cysteine 298, is involved in proton delivery to the active site; therefore it is crucial for substrate supply and product release, since the protons are converted reversibly to molecular hydrogen. In this case, accounting for proton pathways and local delivery engineering, means not only a matter of pH stability and fine regulation, but also of controlling the substrate concentration. We are also pursuing the same SSM strategy on other active site positions. The results of the focused approach on the conserved residue Cys 298, the final amino acid of a proton transfer chain to the active H-cluster [124] and believed to relay proton to the dithiolate bridging group that funnel them to the distal Fe, are reported in a recent publication [55]. Upon saturation mutagenesis with the NNK codon, a colorimetric screening performed on colonies allowed to reach 99.8% coverage of the library. Clones containing an active enzyme (with a detection threshold of 14% of original WT activity) were identified resulting in selection of only WT revertants or Cys-to-Asp mutants. The C298D variant shows a retained activity of 50%, which is interesting since the Cys residue is fully conserved in evolution, and therefore novel mutational spaces were explored, attesting that Asp can functionally replace Cys in proton relay and is structurally compatible (Figure 11). 

The frequency of WT revertants and Asp mutants matched the expected value on the basis of encoding codon frequency. To confirm the library completeness, selected clones were sequenced, showing a good and balanced codon randomization [55].

The SSM approach reported on [NiFe]-hydrogenase [122], performed in combination with directed evolution techniques such as error-prone PCR and shuffling is, as a matter of fact, the first random protein engineering of a hydrogenase. This work targeted the large subunit (HycE) of *Escherichia coli* hydrogenase 3. Hydrogenase 3 is responsible for synthesizing hydrogen from 2H^+^ and 2e^−^ within the supramolecular complex of formate hydrogen lyase (FHL), that also contains a formate dehydrogenase-H for forming 2H^+^, 2e^−^, and CO_2_ from HCOOH: the overall FHL catalyses therefore hydrogen production from formate. A C-terminal truncated variant of HycE, generated by this combined random and SSM approach, showed increased hydrogen production by 30-fold. 

Formate processing enzymes other than the cited subunit of *E. coli* hydrogenase are also relevant for the energy and sustainable process issues in their reversible activity of CO_2_ conversion. The possibility of CO_2_ sequestering and conversion of formate to methanol and methane is an intriguing perspective for research and applications [130]. Also the formate/CO_2_ conversion is coupled to reduction of NAD^+^ to NADH. Formate dehydrogenase from the yeast *Candida boidinii* catalyses the reaction with a selectivity for NAD^+^ only, while NADP^+^ is not recognized as a productive cofactor for the redox reaction and only gives minimal activity. SSM applied to two specific residues, Asp195 and Tyr196, of the dinucleotide-binding region, allowed an improvement in catalytic efficiency with NADP^+^ of the order of 10^7^ [131]. The selected variant Asp195Gln/Tyr196His is relevant for cofactor recycling systems with specificity for NADPH, preferred in enzymes such as cytochrome P450 monooxygenases that are largely applied in industry. The recovery of reduced cofactor is basically the natural strategy for storing solar energy in photosynthetic and chemical energy in chemosynthetic organisms, and therefore the control of biocatalysts performance in this reaction is a step forward in the direction of exploiting and mimicking nature in a sustainable manner.

## 4. Conclusions

The huge number of successful applications of SSM methods to enzymes reported in the last years underlines the feasibility of a semi-random approach to enzyme engineering. The results in activity, specificity and stability enhancement obtained are in several respects more cost-effective and less time-consuming than their counterparts, purely based on random approach and directed evolution. A factor of about ten, comparing enhancement of 20–50 fold by directed evolution and up to 700 for SSM, put SSM far ahead of fully randomized methods achievement-wise. In addition, the number of screened variants required for sound library coverage is generally 2–3 orders of magnitude smaller, allowing for application of more specific screening methods, able to precisely select the desired feature. Generating small and smart libraries is certainly a common and important goal also for the random directed evolution approach. The positive and negative results of both strategies in this direction can give important inputs and shared benefits. The drawback of SSM, *i.e*., the required knowledge of structural data, is becoming less relevant given the increasingly available 3D models that can be calculated by homology with existing structural data and/or *ab-initio* modeling methods. These indirect structural data might not provide details of mechanisms and functions, but they are very indicative for intelligent planning of experimental approaches in SSM. Therefore, at least for technical enzymes, the SSM methods can be foreseen playing a major role in enzyme evolution. In combination with site-directed and random approaches, the methods have the potential to make a difference in exploring novel landscapes for biocatalysts most ambitious refinement, enhancement and application. The challenges remain in the development of biocatalysts performing entirely novel activities. In this respect, the importance of information in the details of mechanism of natural and successfully engineered enzymes is crucial. The role played by rational site-directed mutagenesis in elucidating mechanism and substrate specificity has been of paramount importance. A very recent review focused on an important class of enzymes foresees a similarly important role for SSM [132].

The next generation of engineered biocatalysts can certainly reach unprecedented performances [133] and this can be achieved due to the choices available to scientists to select among different strategies, whose advantages and limitations have to be carefully balanced. The versatility of SSM and the various modifications of this general approach, together with the chance to combine with other strategies, equip protein engineers with an already powerful toolbox. How to apply the tools is not simple to rationalize or give rules for, but this is certainly the undefined area that must remain open, in which scientists can propose original experimental design and improved methods. 
